# Signaling intact membrane-bound IL-15 enables potent anti-tumor activity and safety of CAR-NK cells

**DOI:** 10.3389/fimmu.2025.1658580

**Published:** 2025-09-30

**Authors:** Xiaodi Xu, Peiyu Cao, Meng Wang, Yan Wan, Shuwen Sun, Yuxin Chen, Yilin Liu, Tong Su, Ge Gao, Xinze Liu, Weixiang Zhong, Xi Chen, Xiaoyuan Lu, Buze Chen, Junnian Zheng, Gang Wang, Huizhong Li

**Affiliations:** ^1^ Cancer Institute, Xuzhou Medical University, Xuzhou, Jiangsu, China; ^2^ Center of Clinical Oncology, The Affiliated Hospital of Xuzhou Medical University, Xuzhou, Jiangsu, China; ^3^ Jiangsu Center for the Collaboration and Innovation of Cancer Biotherapy, Xuzhou Medical University, Xuzhou, Jiangsu, China; ^4^ The Second Clinical Medical School, Nanjing Medical University, Nanjing, Jiangsu, China; ^5^ Department of Obstetrics and Gynecology, The Affiliated Hospital of Xuzhou Medical University, Xuzhou, Jiangsu, China

**Keywords:** CAR-NK cells, immunotherapy, membrane-bound IL-15, solid tumors, safety

## Abstract

**Background:**

Chimeric antigen receptor (CAR)-NK cells are a promising and safe alternative to CAR-T cells. However, the limited persistence *in vivo* restricts their clinical application and sustained therapeutic responses. IL-15 has been extensively used to improve CAR-NK cell persistence and effectiveness. Nevertheless, accumulation of IL-15 might induce uncontrolled proliferation of CAR-NK cells and thus lead to fatal side-effects. Therefore, it is essential to develop a safe and effective alternative strategy to improve the persistence and anti-tumor activity of CAR-NK cells.

**Methods:**

A signaling intact membrane-bound IL-15 (mbIL-15) was designed by fusing IL-15 and full-length IL-15Rα and was systematically compared with secretory IL-15 (sIL-15) in a B7H3-targeting CAR-NK cell system regarding their functionality and safety through various *in vitro* and *in vivo* experiments.

**Results:**

Both expression of sIL-15 or mbIL-15 significantly enhanced the proliferation by activating STAT5 and improved anti-tumor activity of CAR-NK cells *in vitro* and *in vivo*. Although CAR-NK cells with sIL-15 quickly eliminated intraperitoneal ovarian cancer, the mice experienced severe consequences, including dysregulated CAR-NK cell expansion, intense inflammatory responses, and irreversible organ damages. In contrast, CAR-NK cells carrying mbIL-15 showed moderate cell proliferation and potent tumor killing activity without observable adverse effects in both local treatment and systemic administration models.

**Conclusion:**

Head-to-head comparative studies demonstrated that signaling intact mbIL-15 significantly improved therapeutic efficacy and safety of CAR-NK cells compared to sIL-15, which provided preclinical evidence for future clinical development.

## 1 Introduction

Natural killer (NK) cells are a vital part of the innate immune system. NK cells can eliminate tumor cells independent of antigen stimulation, which is crucial for immunological surveillance and eradicating cancer (1, 2). Moreover, NK cells can be derived from allogeneic sources without inducing graft-versus-host disease (GVHD) (3, 4). Therefore, NK cells are generally considered more suitable than T cells to develop universal, off-the-shelf immunocyte products. Chimeric antigen receptor (CAR) arming NK cells retain their typical activation and inhibition receptors and exert more potent anti-tumor effect (5). They can discriminate between healthy and malignant cells by balancing activation and inhibition signals, thereby minimizing the risk of recurrence and off-target side-effects (6). Transfusion of CAR-NK cells have been shown great clinical effectiveness without causing severe cytokine release syndrome (CRS) or neurotoxicity in clinical trials (7, 8). However, the limited persistence of CAR-NK cells *in vivo* restricts their duration and sustained anti-tumor responses (9–11).

Cytokines are known essential for maintaining the maturation, activation, proliferation and survival of NK cells, such as IL-2, IL-12, IL-15 *etc* (12). Among them, IL-15 has been broadly studied and found to enhance the survival and expansion of CAR-NK cells (13–15). Despite the greater potency, bioavailability, and stability of the IL-15-IL-15Rα complex compared to recombinant soluble IL-15 for systemic administration (16, 17), incorporating IL-15 encoding gene into the CAR construct is still the preferred strategy to enhance CAR-NK cell function in preclinical mouse models and clinical trials (15, 18, 19).

The clinical trial using anti-CD19 CAR-NK cells that express secretory IL-15 (sIL-15) demonstrated promising results in B-cell lymphoid malignancies (19). However, preclinical studies found that CAR-NK cells engineered to co-express sIL-15 were associated with premature mortality in an immunodeficient mouse model with human MV-4–11 acute myeloid leukemia (AML) cells (20). Notably, the serum level of IL-15 in these mice exceeded 1000 pg/mL. These results indicate that CAR-NK cells carrying sIL-15 might pose significant safety risks. Therefore, it is essential to develop a safe and effective alternative strategy to improve the persistence and anti-tumor activity of CAR-NK cells.

Herein, a signaling intact membrane-bound IL-15 (mbIL-15) was designed by fusing IL-15 and full-length IL-15 receptor with a G4S linker and tested in a B7H3 targeting CAR system. We systematically compared CAR-NK cells co-expressing mbIL-15 and sIL-15 regarding their functionality and safety through various *in vitro* and *in vivo* experiments. *In vitro* experiments indicated that either co-expression of sIL-15 or mbIL-15 activated downstream STAT5 signaling, which was previously proved to regulate the proliferation and survival of CAR-NK cells. Moreover, both forms of IL-15 significantly enhanced the anti-tumor activity of CAR-NK cells. In tumor-bearing mouse models, CAR-NK cells co-expressing sIL-15 rapidly eliminated ovarian cancer cells in the peritoneal cavity of mice. However, the continuous secretion of IL-15 caused uncontrolled proliferation of CAR-NK cells, leading to severe inflammatory responses and damage to major organs, which ultimately resulted in early death of the mice. In contrast, mbIL-15 armored CAR-NK cells exhibited moderate cell proliferation and effective tumor-killing activity through both intraperitoneal and intravenous delivery, significantly extending the survival of mice without observable adverse effects. In conclusion, co-expression of the signaling intact mbIL-15, compared to sIL-15, significantly enhanced the therapeutic efficacy and safety of CAR-NK cells, providing valuable preclinical evidence for future clinical CAR-NK cell product development.

## 2 Methods

### 2.1 Cell culture

Peripheral blood mononuclear cells (PBMCs) from healthy donors were isolated using density gradient centrifugation. The human ovarian cancer cell line SKOV-3, human lung cancer cell line A549, human prostate cancer cell line DU145, human liver cancer cell line Huh7, and human embryonic kidney cell line 293T were obtained from the Cell Bank of the Chinese Academy of Sciences. DU145 and Huh7 were cultured in MEM medium containing 10% fetal bovine serum (FBS) and 1% penicillin/streptomycin (100 µg/mL). A549 and SKOV-3 were cultured in DMEM medium with 10% FBS and 1% penicillin/streptomycin. 293T was cultured in IMDM medium with 10% FBS and 1% GlutaMax (Thermo Fisher Scientific, USA). Activated NK cells and CAR-NK cells were cultured in GT-T551 H3 (TAKARA, Japan) medium containing 500 IU/mL recombinant human interleukin-2 (rhIL-2) (Cat. 101-02, PrimeGene, Shanghai). All cells were incubated at 37°C in a humidified atmosphere with 5% CO_2_.

### 2.2 CAR constructs

B7H3 CAR vector was designed, incorporating a B7H3-specific single-chain antibody (scFv), a CD8α hinge and transmembrane domain, CD28 and CD3ζ intracellular signaling domains (*B7H3-CAR*). The sequence of human secretory IL-15 (sIL-15) was integrated into the B7H3 CAR structure via a Thosea asigna virus 2A (T2A) peptide, constructing a CAR plasmid vector that co-expressed sIL-15 (*B7H3-sIL15-CAR*). The full-length human IL-15 was fused with the full-length IL-15Rα sequence using a flexible linker peptide G4S (GGGGS × 3) to create a membrane-bound IL-15 (mbIL-15) sequence. This mbIL-15 sequence was then inserted into the T2A peptide sequence of the B7H3 CAR construct, resulting in a CAR plasmid vector co-expressing mbIL-15 (*B7H3-mbIL15-CAR*).

### 2.3 Retrovirus packaging

To generate retroviral particles carrying CAR for NK transduction, MSCV-CAR vector and pCL-Ampho helper plasmid were co-transfected into 293T cells using the GeneJuice transfection reagent (Cat. 70967, Merck Millipore, Germany) according to the manufacture’s instruction. Supernatant containing the CAR-carrying retroviruses were collected at 48 and 72 hours post transfection and filtered with 0.45μm filters and stored at -80°C.

### 2.4 Preparation of NK and CAR-NK cells

NK cells were expanded using a feeder-cell-free method. Briefly, peripheral blood mononuclear cells (PBMCs) from healthy donors were isolated by density gradient centrifugation using Lymphoprep™ (Cat. 07851, STEMCELL, Canada) and resuspended in GT-T551 H3 medium (TAKARA, Japan) supplemented with 500 IU/ml recombinant human IL-2 (Cat. 101-02, PrimeGene, China) and 1 μg/ml OK432 (T&L, China). The cells were seeded into anti-CD16 antibody (Beckman Coulter, USA)-precoated flasks and cultured at 37°C under 5% CO_2_ for 3 days before being transferred to conventional flasks. Throughout the 14-day expansion period, the cells were subcultured every 2–3 days and maintained at a density of approximately 1×10^6^ cells/mL in fresh medium. The percentage of NK cells was determined by flow cytometry after staining with FITC-conjugated anti-human CD3 antibody (Cat. 981002, Clone SK7, BioLegend, USA) and APC-conjugated anti-human CD56 antibody (Cat. 981204, Clone 5.1H11, Biolegend, USA).

Before NK transduction, non-treated tissue culture 24-well plates were pre-coated with 7μg/mL of retronectin (Cat. T100B, Takara, Japan) at 4°C overnight. Twenty-four hours later, retronectin were removed from the 24-well plates and then were washed once with PBS. 1 mL of retroviral supernatant were added into each well and centrifuged at 2000 g for 90 minutes at 32°C. After removal of the supernatant, 5×10^5^ of cytokine-activated NK cells in fresh culture medium containing 500U/mL of IL-2 at day 6 were seeded into each well and centrifuged at 1000 g for 10 minutes. After three days, part of CAR-NK cells were collected for CAR detection using flow cytometer. On day 12-14, CAR-NK cells were used for *in vitro* and *in vivo* experiments.

### 2.5 Carboxyfluorescein-diacetate-succinimidyl esters (CFSE)

CAR-NK cells were cultured in medium without FBS and rhIL-2 for 24 hours. Subsequently, Cells were collected and washed thoroughly with PBS containing 0.1% FBS. Resuspend the cell pellet in 500 μL of 1.5 mM CFSE working solution (Cat. C34570, Invitrogen, USA) and incubate in the dark at room temperature for 10 minutes. Following this, add 500 μL of FBS and incubate the cells in a 37°C water bath for an additional 10 minutes. After incubation, wash the cells twice with PBS containing 2% FBS. Obtain a small aliquot of cells for flow cytometry analysis to measure CFSE fluorescence as a control. After 72 hours, CFSE fluorescence was analyzed by flow cytometry to evaluate their *in vitro* proliferation capabilities.

### 2.6 Multiplex cytokine quantification assay and ELISA

1×10^5^ CAR-NK cells were cultured in 1 mL of complete medium without any cytokines at 37°C, 5% CO_2_ for 24 hours later, the cell culture supernatant was collected, and the concentration of human IL-15 in the supernatant was determined using Human IL-15 Precoated ELISA Kit (Cat.1111502, Dakewe, Shenzhen).

2×10^5^ CAR-NK cells were co-cultured with tumor cells in 1 mL of complete medium without any cytokines at an E:T ratio of 1:1 for 24 h at 37°C, 5% CO_2_. The concentrations of TNF-α, granzyme B and IFN-γ in the co-culture supernatant were determined using Human TNF-α Precoated ELISA Kit (Cat.1117202, Dakewe, Shenzhen), Human granzyme B Precoated ELISA Kit (Cat.1118502, Dakewe, Shenzhen) and Human IFN-γ Precoated ELISA Kit (Cat.1110002, Dakewe, Shenzhen) ELISA kit. All procedures were performed following the manufacturer’s protocols.

### 2.7 CD107a degranulation assay

5×10^4^ of SKOV-3 cells were seeded into a 96-well plate. Add NK or CAR-NK cells to each well at an E:T ratio of 5:1. Right afterward, add 1 μL of PE-Cy7 anti-human CD107a antibody (Cat. 328617, Clone H4A3, BioLegend, USA) to each well. To prevent protein secretion and degradation of internalized CD107a, brefeldin A (2 μg/mL) are added after 1 h of incubation. Following an additional 5 h incubation, collect the cells and stain with APC anti-human CD56 (Cat. 981204, Clone 5.1H11, Biolegend, USA) for 30 minutes at 4°C in the dark. The percentage of CD107a^+^ NK cells was determined by flow cytometry.

### 2.8 Cytotoxicity assay

The cytotoxicity of NK or CAR-NK cells on SKOV-3, Huh7, DU145, and A549 cells was evaluated using the xCELLigence Real-Time Cell Analysis (RTCA) system (ACEA Biosciences, USA). 50 μL of medium was added to E-Plates 16 to measure background values. The tumor cells were seeded and cultured at a density of 1×10^4^ cells per well. After the cell index cell index of each well was close to 1, NK or CAR-NK cells were added to the corresponding well at an E:T ratio of 1:8. Electrical impedance was measured every 15 minutes. In the RTCA cytotoxicity assays, cell status was monitored in real-time via electrical impedance, expressed as the Cell Index (CI), a unitless parameter. For quantitative analysis of cytotoxicity, the Normalized Cell Index (NCI) was calculated relative to the CI value at the time point immediately prior to the addition of effector cells following the manufacture’s instruction. All analyses were performed using the integrated RTCA Data Analysis Software Package (version 1.0, ACEA Biosciences, USA).

### 2.9 Western blot assay

NK or CAR-NK cells were cultured in the presence or absence of rhIL-15 for 24 h. Subsequently, the cells were collected and washed twice with cold PBS. Cells were lysed on ice for 30 minutes using RIPA lysis buffer containing a protease inhibitor. Protein concentration was determined using a BCA protein assay kit (Cat. 981204, Thermo Fisher Scientific, USA).

Equal amounts of protein samples from each group were separated by 10% SDS-PAGE and transferred to a nitrocellulose membrane (Pall, USA). The membrane was blocked with 5% non-fat milk in TBST at room temperature for 1 hour. The NC membrane was incubated overnight at 4 °C with primary antibodies diluted in 5% non-fat milk against STAT5 (Cat. 25656, 1:1,000, CST, USA), p-STAT5 (Thr694) (Cat. 4322, 1:1,000, CST, USA), AKT (Cat. A11016, 1:1,000, ABclonal, USA), p-AKT (Ser473) (Cat. AP0140, 1:1,000, ABclonal, USA), β-actin (Cat. 66009-1-Ig, 1:5,000, Proteintech, USA), and GAPDH (Cat. 5174, 1:1,000, CST, USA). The NC membrane was washed three times with TBST. It was then incubated with HRP-conjugated goat anti-rabbit secondary antibody (Cat. SA00001-2, 1:10,000, Proteintech, USA) or HRP-conjugated goat anti-mouse secondary antibody (Cat. SA00001-1, 1:10,000, Proteintech, USA) diluted in 5% non-fat milk at room temperature for 1 hour.

Immunoreactive bands were developed using a chemiluminescence substrate (NCM Biotech, China) and visualized with a Tanon-5200 chemiluminescence imaging system (Tanon, China).

### 2.10 Xenograft mouse model

Five-week-old female NOD/ShiLtJGpt-*Prkdc* ^em26^ *Il2rg* ^em26^/Gpt (NCG) mice were obtained from Nanjing GemPharmatech (Nanjing, China) and maintained under specifc pathogen-free (SPF) conditions. The mice were housed at a temperature of 23 ± 1 °C, relative humidity of 40-70%, light intensity of 20 lx, and a 12 h/12 h light-dark cycle with free access to food and water, with 5 mice per cage. After 7 days of acclimatization, the experiments were initiated. All *in vivo* procedures were performed under inhalation anesthesia induced with 2–3% isoflurane and maintained with 1–2% isoflurane in oxygen. All animal procedures and protocols were approved by the Experimental Animal Ethics Committee of Xuzhou Medical University (202009A055), and implemented in accordance with the Xuzhou Medical University Laboratory Animal Care and Use Guide.

For ovarian cancer models, each mouse was injected intraperitoneally (*i.p.*) with 5×10^5^ luciferase-GFP labeled SKOV-3 cells. Five days later, mice were injected *i.p*. with D-luciferin (150 mg/kg, Cat. 710503ES08, Yeasen, Shanghai) and imaged 10 minutes later. Tumor burdens were monitored by total bioluminescent flux using a living imaging system (NightOWL II LB983, Berthold, Germany). After tumor establishment, mice were randomly assigned to groups using a random number sequence (5 mice per group). The grouped mice received treatment via intraperitoneal or intravenous injection of NK or CAR-NK cells. Tumor progression during treatment was monitored using the small animal *in vivo* imaging system. IndiGo software (Berthold, Germany) was used to analyze the total bioluminescence flux.

Peripheral blood from mice was stained with FITC-anti-human-CD3 (Cat. 981002, Clone SK7, BioLegend, USA), APC anti-human CD56 (Cat. 981204, Clone 5.1H11, Biolegend, USA), recombinant human B7-H3 Fc protein (Cat. 10472-B3-050, R&D system, USA) and PE-Goat anti-Human IgG Fc antibody (Cat. 12-4998-82, eBioscience, USA), and Count Beads (Cat. 424902, Biolegend, USA) were added for counting. The frequency and number of CAR-NK cells in the peripheral blood were analyzed using flow cytometry. Following the experimental procedures, mice were euthanized by exposure to 100% CO_2_ in a sealed chamber at a flow rate that displaced 30-50% of the chamber volume per minute. The concentration of human IL-15 in mouse serum was quantified using a Human IL-15 Precoated ELISA Kit (Cat.1111502, Dakewe, Shenzhen). The major organs of the mice were stained with hematoxylin and eosin.

### 2.11 Flow cytometry

The purity of NK cells was assessed using FITC-anti-human-CD3 antibody (Cat. 981002, Clone SK7, BioLegend, USA) and APC-anti-human-CD56 antibody (Cat. 981204, Clone 5.1H11, BioLegend). The expression levels of the natural cytotoxicity receptor (NCR) and natural killer receptor G2D (CD314) on NK cells were evaluated using PerCP/Cyanine5.5-anti-human-CD314 antibody (Cat. 320817, Clone 1D11, BioLegend), PerCP/Cyanine5.5-anti-human-CD337 (NKp30) antibody (Cat. 325215, Clone P30-15, BioLegend), PE-anti-human-CD336 (NKp44) antibody (Cat. 325107, Clone P44-8, BioLegend), and APC/Cyanine7-anti-human-CD335 (NKp46) antibody (Cat. 331949, Clone 9E2, BioLegend). The expression levels of B7H3 (CD276) on SKOV-3, Huh7, DU145, and A549 cells were quantified using PE-anti-human-CD276 (B7H3) antibody (Cat. 331606, Clone DCN.70, BioLegend) along with an appropriate isotype control (Isotype Ctrl Antibody κ, BioLegend). CAR expression was assessed using recombinant human B7-H3 Fc protein (Cat. 10472-B3-050, R&D system, USA) and PE-Goat anti-Human IgG Fc antibody (Cat. 12-4998-82, eBioscience, USA). Cell apoptosis was evaluated using an Annexin V-FITC/PI dual-staining apoptosis detection kit (KeyGEN BioTECH, Jiangsu). All analyses were performed on a FACSCanto II flow cytometer (Becton, Dickinson and Company, USA), and data were analyzed using FlowJo software (Version 10).

### 2.12 Statistical analysis

All data are expressed as mean ± standard deviation (SD), unless otherwise specified. Statistical analyses were performed using GraphPad Prism 9.0.0 software. The normality of data distribution was verified using the Shapiro-Wilk test before applying parametric tests. Differences between two independent samples were assessed using an unpaired Student’s t-test, while comparisons among multiple groups were conducted using one-way analysis of variance (ANOVA). For ANOVA that yielded significant results, multiple comparisons were performed using Tukey’s honest significant difference (HSD) *post hoc* test. Survival analysis was performed using the log-rank (Mantel-Cox) test. A value of *p* < 0.05 was considered statistically significant for all experiments.

## 3 Results

### 3.1 Ectopic expression of IL-15 induces lethal toxicity *in vivo* despite potent anti-tumor activity

IL-15 has been shown to enhance the antitumor activity and longevity of NK cells. To harness this benefit, we incorporated the sequence of human IL-15 (sIL15) into a CAR vector composing by B7H3-specific single-chain variable fragment (scFv), CD8 hinge and transmembrane domains, the intracellular signaling domains of CD28 and CD3ζ with the T2A peptide (**Supplementary Figure 1A**). Flow cytometry assay showed that the transduction efficiency of CAR on NK cells was approximately 80% (**Supplementary Figures 1B, C**). B7H3-sIL15-NK cells secreted significantly higher (*p* < 0.001) level of IL-15 as compared to NK cells and B7H3-NK cells (**Supplementary Figure 1D**). Continuous cell counting revealed that IL-15 expression markedly boosted CAR-NK cell proliferation (**Supplementary Figure 1E**). Flow cytometry confirmed the high expression of the B7H3 antigen on SKOV-3, A549, Huh7, and DU145 cells (**Supplementary Figure 1F**). Additionally, the cytotoxicity of CAR-NK cells against the B7H3^+^ tumor cell lines at an effector-to-target ratio of 1:8 was assessed by Real-Time Cell Analysis (RTCA). Compared to untransduced NK cells, CAR-NK cells demonstrated more potent antitumor activity. Moreover, ectopic expression of IL-15 further enhanced the cytotoxic effects of CAR-NK cells (**Supplementary Figure 1G**). Therefore, overexpression of IL-15 enhances CAR-NK cell proliferation. and tumor killing effect.

**Figure 1:**
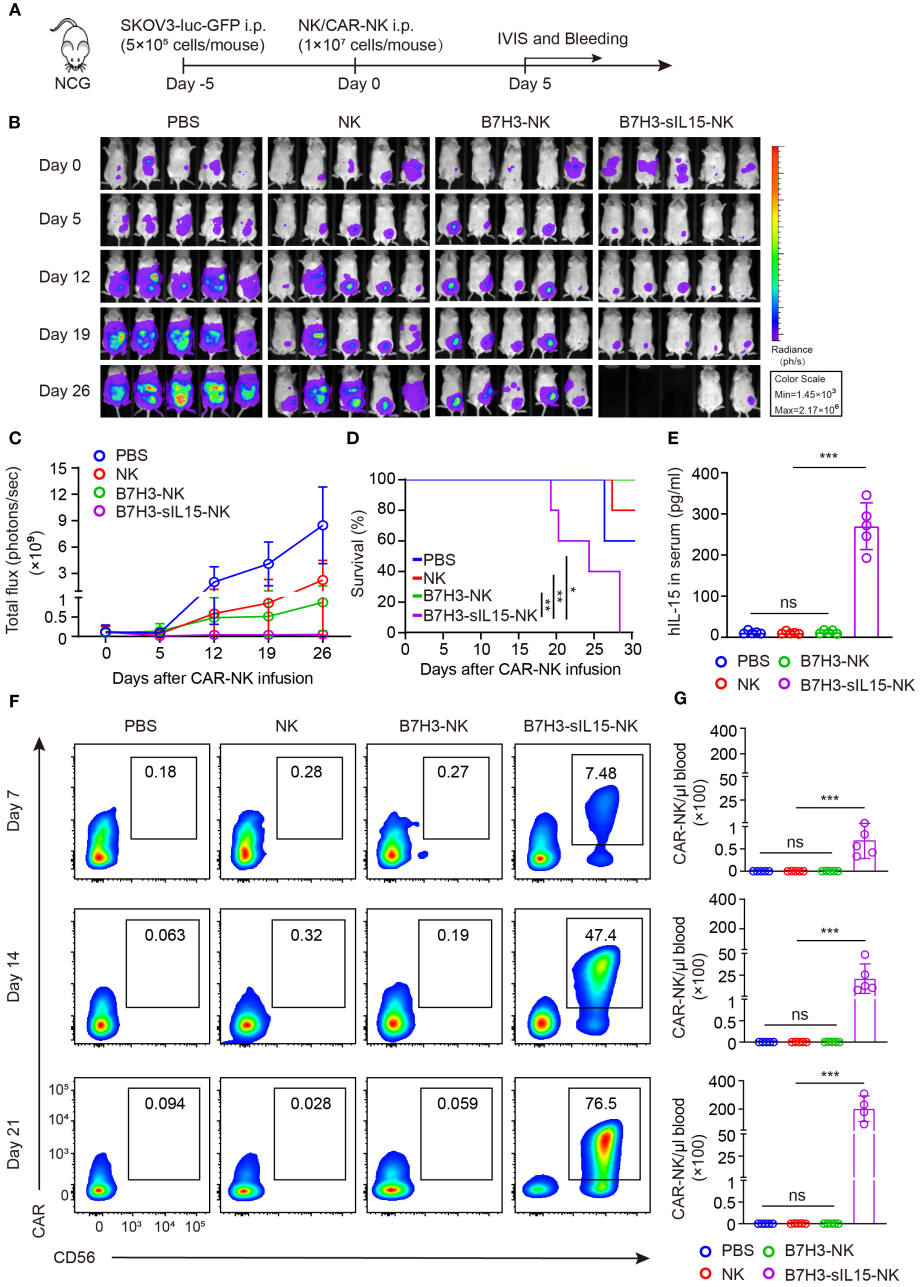
sIL-15 promotes the antitumor activity and persistence of B7H3-NK cells *in vivo*; however, it also induces lethal toxicity. **(A)** Schematic diagram of intraperitoneal injection of 1×10^7^ NK, B7H3-NK, or B7H3-sIL15-NK cells for the treatment of luciferase-labeled SKOV-3 intraperitoneal xenograft model. **(B)** Bioluminescent monitoring of luciferase-labeled SKOV-3 tumor growth. **(C)** Quantification of the bioluminescence data shown in **(B)**. **(D)** Kaplan-Meier survival curve of mice in **(B)**. **(E)** Human IL-15 concentration in the plasma of mice at the end of the experiment was measured by ELISA. **(F, G)** Proportion and number of CAR-NK cells in mouse blood were assessed by flow cytometry on days 7, 14, and 21 following intraperitoneal injection with 1×10^7^ NK, B7H3-NK, or B7H3-sIL-15-NK cells. **(F)** Representative flow cytometry results of CAR-NK cells proportion in mouse peripheral blood. **(G)** Statistical graph of CAR-NK cells number in mouse peripheral blood. n=5 mice for each group. One-way analysis of variance was used for comparisons among multiple groups. Survival analysis was performed using the log-rank (Mantel-Cox) test. The data expressed as means ± SD. ns: not significant, * *p* < 0.05, ** *p* < 0.01 and *** *p* < 0.001.

To further evaluate the anti-tumor activity and safety of CAR-NK cells that co-expressing IL-15, an intraperitoneal xenograft model of ovarian cancer was established. First, 5×10^5^ of SKOV-3 cells expressing luciferase were injected intraperitoneally into NCG mice. Five days later, the tumor formation was confirmed by live imaging. Then, the mice were randomly divided into 4 groups and received intraperitoneal injections of PBS, 1×10^7^ NK cells, B7H3-NK cells, or B7H3-sIL15-NK cells respectively (**Figure 1A**). Tumor burden was monitored by measuring tumor bioluminescence at scheduled timepoints. The results showed that CAR-NK cells co-expressing IL-15 could rapidly remove the tumors in the abdominal cavity of mice (**Figures 1B, C**). Mice treated with CAR-NK cells co-expressing sIL-15 died around 20 days after treatment, unlike the other groups (**Figure 1D**). To figure out the potential lethal factors, serum IL-15 and CAR-NK cells in the peripheral blood were measured. High levels of human IL-15 were detected in the serum of mice treated with B7H3-sIL15-NK cells ((**Figure 1E**). Additionally, superabundant and increasing CAR-NK cells in the peripheral blood were found in mice treated with B7H3-sIL15-NK cells on days 7, 14, and 21 (**Figures 1F, G**). Hence, co-expression of sIL-15 would cause uncontrollable growth of CAR-NK cells which might be harmful for mice and leading to lethal toxicity.

### 3.2 Anchoring signaling intact IL-15 and IL-15Rα fusion protein to cell membrane enhanced proliferation and survival of CAR-NK cells and avoided IL-15 secretion

**Figure 2:**
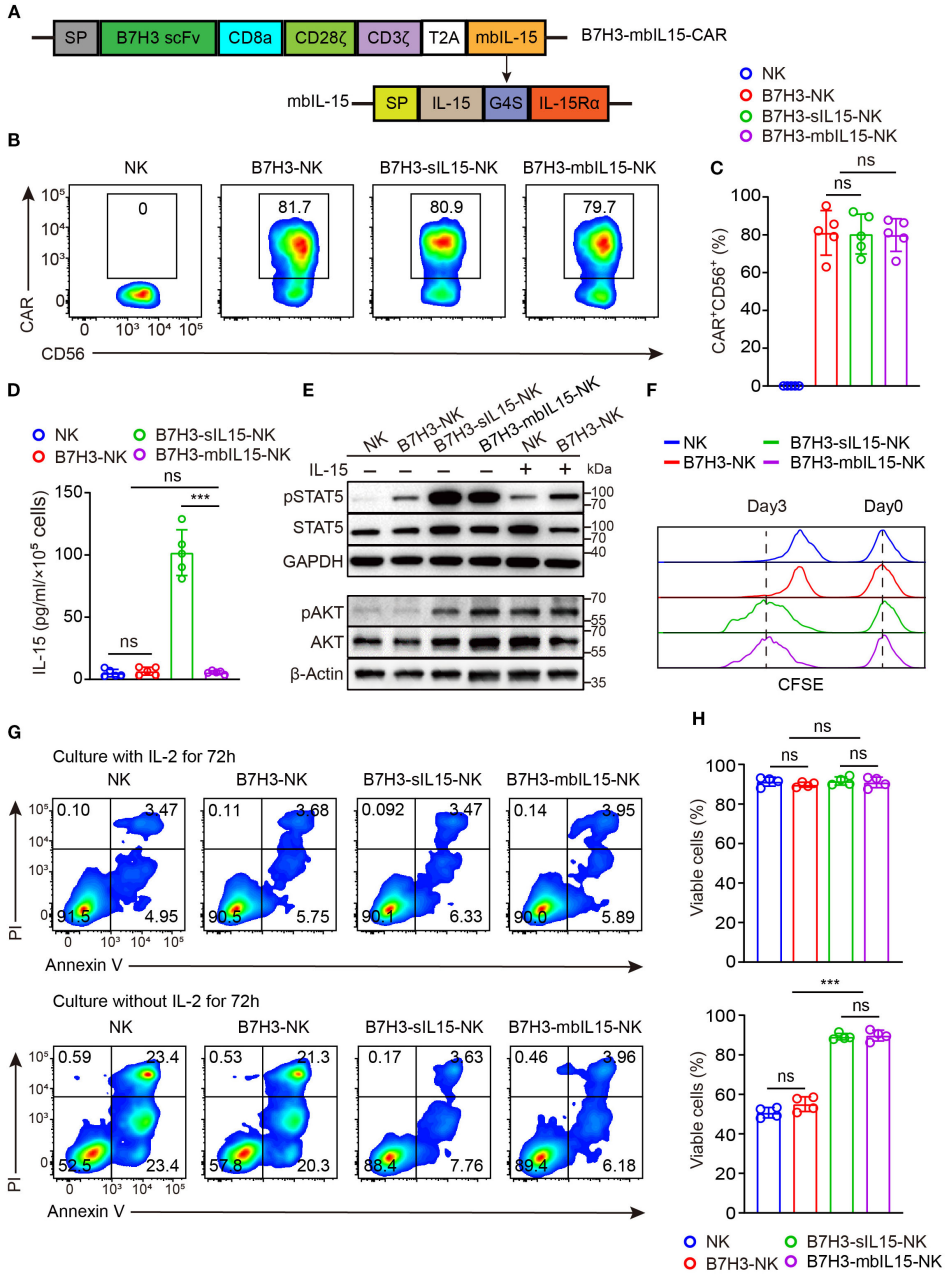
mbIL-15 enhances the proliferation and survival of B7H3-NK cells *in vitro*. **(A)** Structural schema of the B7H3-sIL15-CAR and B7H3-mbIL15-CAR constructs. **(B)** CAR expression in B7H3-NK, B7H3-sIL-15-NK, or B7H3-mbIL15-NK cells was measured by flow cytometry. **(C)** Statistical analysis of the results shown in **(B)**. Data are from 5 independent experiments. **(D)** Secretion of IL-15 in the supernatant of 1 × 10^5^ NK, B7H3-NK, B7H3-sIL15-NK, or B7H3-mbIL15-NK cells was measured by ELISA (n=5). **(E)** Phosphorylation levels of STAT5 and AKT in NK, B7H3-NK, B7H3-sIL15-NK, or B7H3-mbIL15-NK cells were examined by Western blot. **(F)** Proliferation of NK, B7H3-NK, B7H3-sIL15-NK, and B7H3-mbIL15-NK cells was evaluated using CFSE staining. **(G)** Cell viability of NK, B7H3-NK, B7H3-sIL15-NK, or B7H3-mbIL15-NK cells was assessed by flow cytometry. **(H)** Statistical analysis of the results shown in **(G)**. Data are from 4 independent experiments. One-way analysis of variance was used for comparisons among multiple groups. Data are means ± SD. ns: not significant, *** *p* < 0.001.

In the aforementioned animal experiments, the results showed that treating with CAR-NK cells co-expressing sIL-15 increased human IL-15 levels in mouse blood and caused uncontrolled proliferation of CAR-NK cells and mice death. To mitigate side effects from continuous IL-15 secretion, a signaling intact mbIL-15 was designed by fusing IL-15 with the full-length IL-15 receptor alpha (IL-15Rα) using a flexible linker G4S. Subsequently, the mbIL-15 sequences were inserted into the CAR construct with a T2A peptide (**Figure 2A**). The transduction efficiency of CAR-NK cells was not affected by co-expression of mbIL-15, which ranged from 70% to 90% (**Figures 2B, C**). Compared to B7H3-sIL15-NK cells, the culture supernatant of B7H3-mbIL15-NK cells contains nearly undetectable levels of human IL-15 (**Figure 2D**). To evaluate its function, the downstream signaling of STAT5 was detected. The results demonstrated that mbIL-15 could more potently activate STAT5 as well as sIL-15 compared to exogenous IL-15 (**Figure 2E**). Moreover, *ex vivo* culture results showed that limited proliferation and decreased cell viability was observed in NK and B7H3-NK cells without the exogenous cytokine IL-2. Interestingly, co-expression of mbIL-15 or sIL-15 successfully sustained the proliferation and survival of CAR-NK cells (**Figures 2F-H**). Therefore, engineering mbIL-15 as a membrane-anchored fusion protein not only provides constitutive support for CAR-NK cell proliferation and survival but also structurally prevents its secretion, thereby minimizing the risk of systemic toxicity.

### 3.3 mbIL-15 enhanced the antitumor cytotoxicity of CAR-NK cells *in vitro*

**Figure 3:**
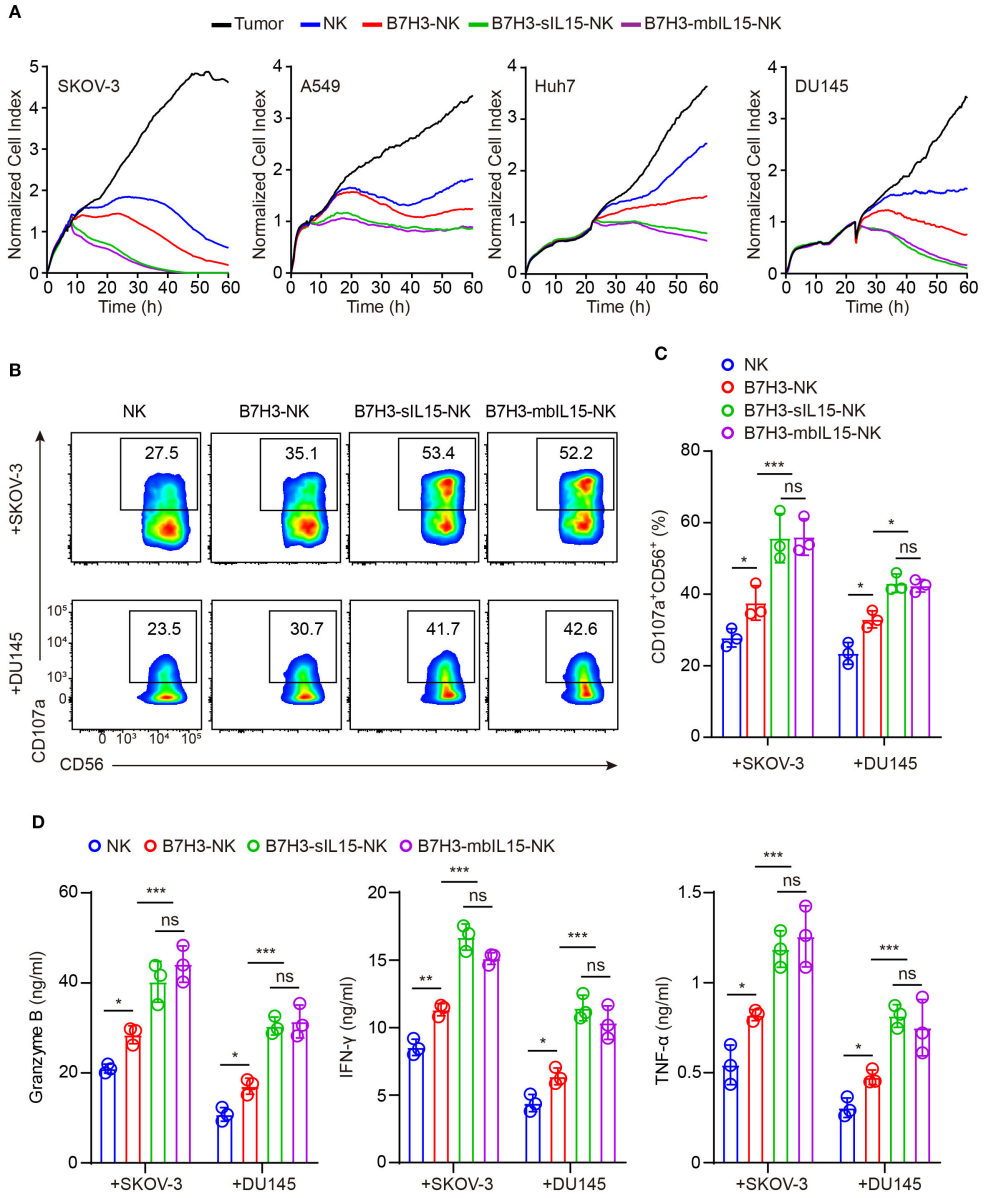
mbIL-15 promotes B7H3-NK cells anti-tumor effect *in vitro*. **(A)** Cytotoxicity of NK, B7H3-NK, B7H3-sIL15-NK or B7H3-mbIL15-NK cells against B7H3^+^ tumor cells (SKOV-3, Huh7, DU145, and A549) at an E:T ratio of 1:8 was assessed using RTCA. **(B)** CD107a expression in NK, B7H3-NK, B7H3-sIL15-NK or B7H3-mbIL15-NK cells was measured by flow cytometry following a 5-hour co-culture with B7H3^+^ tumor cells (SKOV-3 and DU145) at an E:T ratio of 5:1. **(C)** Statistical analysis of **(B)**. **(D)** Secretion of Granzyme B, IFN-γ, and TNF-α by 2×10^5^ NK, B7H3-NK, B7H3-sIL15-NK or B7H3-mbIL15-NK cells was measured by ELISA after a 24-hour co-culture with B7H3^+^ tumor cells (SKOV-3 and DU145) at an E:T ratio of 1:1. Data are from 3 independent experiments. One-way analysis of variance was used for comparisons among multiple groups. Data are means ± SD. ns: not significant, * *p* < 0.05, ** *p* < 0.01 and *** *p* < 0.001.

Subsequently, the cytotoxicity of CAR-NK cells armoring with mbIL-15 against solid tumors was measured. NK cells, B7H3-NK cells, B7H3-sIL15-NK cells, and B7H3-mbIL15-NK cells were co-cultured with B7H3-positive tumor cells at an effector-to-target ratio of 1:8, and their cytotoxicity was assessed using Real-Time Cell Analysis. The results indicated that co-expression either mbIL-15 or sIL-15 increased the cytotoxicity of CAR-NK cells against solid tumors *in vitro* (**Figure 3A**). Additionally, cytotoxic degranulation and cytokine release assays were performed to evaluate the function of CAR-NK cells. After co-culturing with SKOV-3 cells and DU145 cells, we found that overexpression of either mbIL-15 or sIL-15 significantly increased the membrane expression of degranulation protein CD107a (**Figures 3B, C**). Furthermore, there was a notable increase in the levels of antitumor factors such as IFN-γ, Granzyme B, and TNF-α in both mbIL-15 and sIL-15 armed CAR-NK cells. (**Figure 3D**).

### 3.4 Armoring with mbIL-15 improved the therapeutic efficacy and safety of CAR-NK cells for cancer treatment

**Figure 4:**
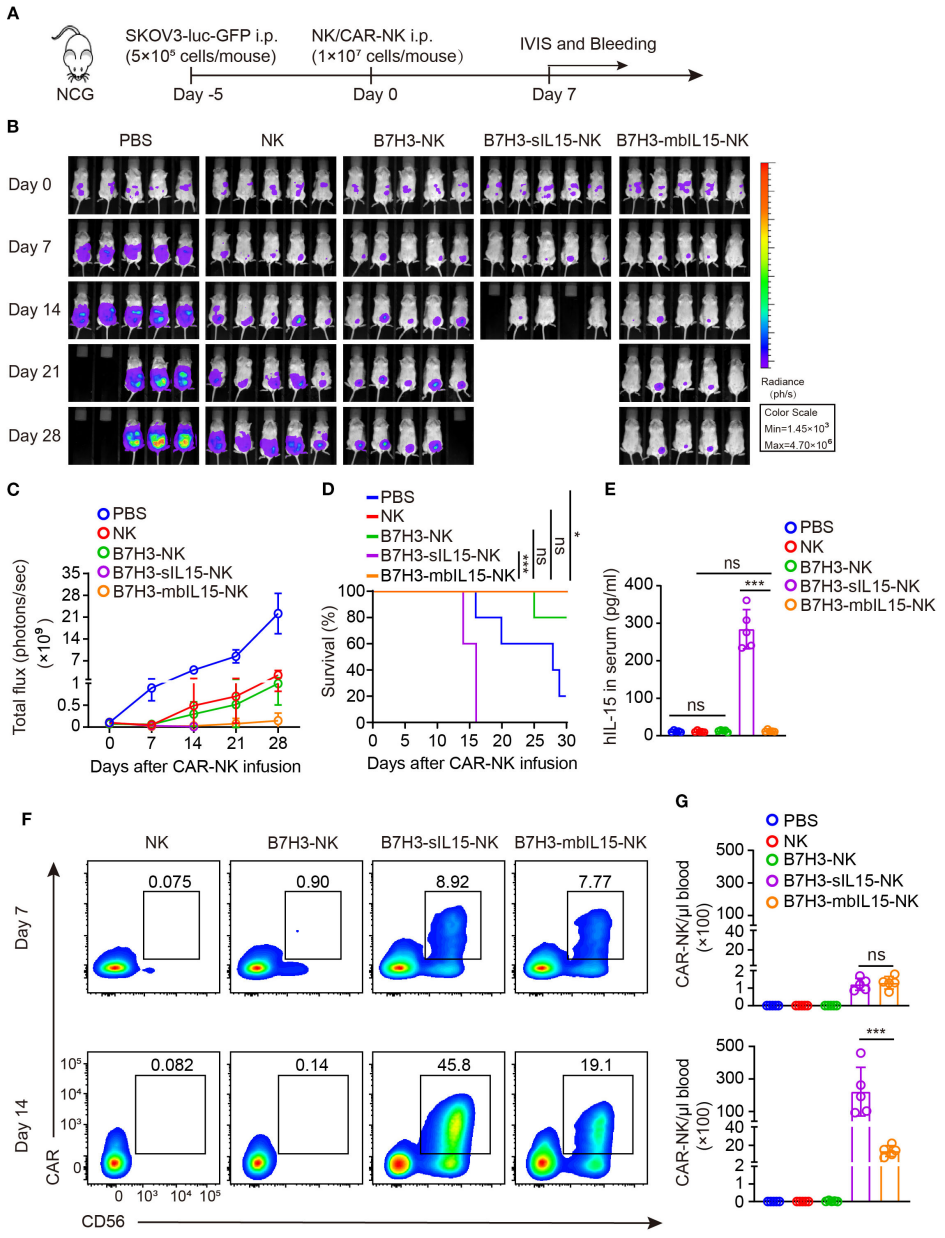
mbIL-15 enhances the antitumor activity and persistence of B7H3-NK cells *in vivo*. **(A)** Schematic diagram of intraperitoneally administrated NK, B7H3-NK, B7H3-sIL15-NK or B7H3-mbIL15-NK cells therapy in a luciferase-labeled SKOV-3 ovarian cancer xenograft model. **(B)** Bioluminescent monitoring of luciferase-labeled SKOV-3 cells. n=5 mice for each group. **(C)** Quantification of the bioluminescence data shown in **(B)**. **(D)** Kaplan-Meier survival curve of mice in **(B)**. **(E)** Human IL-15 concentration in the plasma of mice at the end of the experiment was measured by ELISA. **(F, G)** Proportion and number of CAR-NK cells in mouse blood were assessed by flow cytometry on days 7 and 14 following intraperitoneal injection with 1×10^7^ NK, B7H3-NK, B7H3-sIL15-NK or B7H3-mbIL15-NK cells. **(F)** Representative flow cytometry results of CAR-NK cells proportion in mouse peripheral blood. **(G)** Statistical graph of CAR-NK cells number in mouse peripheral blood. One-way analysis of variance was used for comparisons among multiple groups. Survival analysis was performed using the log-rank (Mantel-Cox) test. The data expressed as means ± SD. ns: not significant, * *p* < 0.05, ** *p* < 0.01 and *** *p* < 0.001.

To evaluate the antitumor activity, persistence and safety of CAR-NK cells armed by mbIL-15, the mouse xenograft cancer model was established to mimic the peritoneal dissemination found in most patients with advanced ovarian cancer (**Figure 4A**). After treatment, intraperitoneal tumors in mice were monitored weekly by measuring bioluminescence. Consistent with previous results, all treatments showed potent antitumor activity (**Figures 4B-D**). However, NK cells and B7H3-NK cells exhibited only temporary antitumor activity after intraperitoneal injection. In contrast, CAR-NK cells co-expressing mbIL-15 or sIL-15 significantly prevented tumor growth in mice. As expected, treatment with CAR-NK cells armed by sIL-15 resulted in premature death of mice 2–3 weeks later after infusion instead of mbIL-15 CAR-NK cells (**Figures 4B-D**). Additionally, human IL-15 was undetectable in the serum of mice treated with B7H3-mbIL15-NK cells, unlike in those treated with B7H3-sIL15-NK cells (**Figure 4E**). The frequency and number of CAR-NK cells in the blood of mice was measured on days 7 and 14 after treatment using flow cytometry. The results indicated that co-expression of mbIL-15 effectively enhanced the persistence and survival of CAR-NK cells *in vivo* over the 14-day observation period (**Figures 4F, G**).

In addition, to assess the safety of CAR-NK cells that co-expressing mbIL-15, the major organs of mice were collected at the end of the experiment and performed paraffin embedding and H&E staining. There were no significant damages to the major organs of spleen, liver, and lungs observed in the mice treated with CAR-NK cells co-expressing mbIL-15. Conversely, treatment with CAR-NK cells carrying sIL-15 caused severe inflammatory responses and significant tissue damage in the major organs of mice (**Supplementary Figure 2**). So, overexpression of mbIL-15 is much safer than sIL-15 in CAR-NK cells.

**Figure 5:**
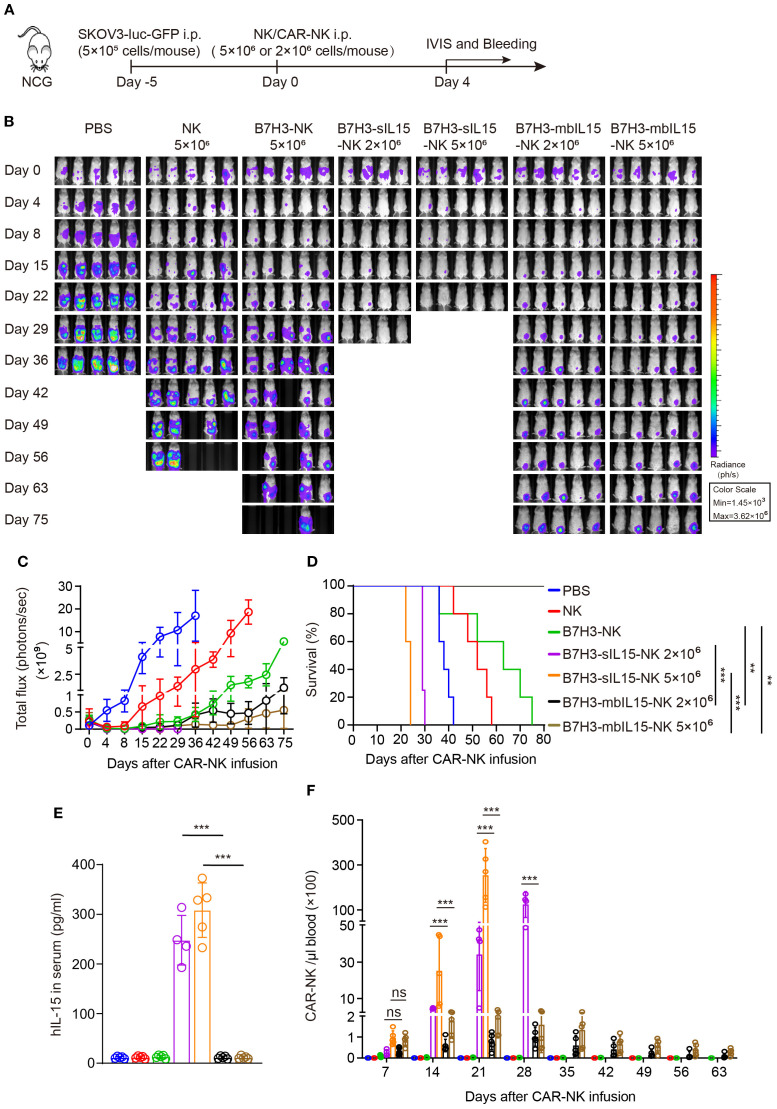
mbIL-15 enhances the therapeutic efficacy and persistence of low-dose B7H3-NK cells *in vivo*. **(A)** Schematic diagram of intraperitoneally administrated 5×10^6^ NK, 5×10^6^ B7H3-NK, 5×10^6^ B7H3-sIL15-NK, 5×10^6^ B7H3-mbIL15-NK, 2×10^6^ B7H3-sIL15-NK or 2×10^6^ B7H3-mbIL15-NK cells therapy in a ovarian cancer xenograft model. **(B)** Bioluminescent monitoring of luciferase-labeled SKOV-3 cells. n=5 mice for each group except 2×10^6^ B7H3-sIL15-NK group (n=4). **(C)** Quantification of the bioluminescence data shown in **(B)**. **(D)** Kaplan-Meier survival curve of mice in **(B)**. **(E)** Human IL-15 concentration in the plasma of mice at the end of the experiment was measured by ELISA. **(F)** Number of CAR-NK cells in mouse blood were assessed by flow cytometry on days 7, 14, 21, 28, 35, 42, 49, 56 and 63 following intraperitoneal injection with 5×10^6^ NK, 5×10^6^ B7H3-NK 5×10^6^ or 2×10^6^ B7H3-sIL15-NK and 5×10^6^ or 2×10^6^ B7H3-mbIL15-NK cells. One-way analysis of variance was used for comparisons among multiple groups. Survival analysis was performed using the log-rank (Mantel-Cox) test. The data expressed as means ± SD. ns: not significant, ** *p* < 0.01 and *** *p* < 0.001.

To further evaluate whether the dosage would affect the side-effects and therapeutic efficacy. In the following *in vivo* experiments, two lower dosages of B7H3-mbIL15-NK and B7H3-sIL15-NK cells were used (**Figure 5A**). Even reducing the treatment dose of CAR-NK cells co-expressing sIL-15 to 2×10^6^, lethal toxicity and premature death of the mice was still observed after infusion for 3 weeks. Interestingly, with a reduced intraperitoneal injection dose 2×10^6^, CAR-NK cells arming with mbIL-15 effectively inhibited tumor progression and significantly prolonged the survival of tumor-bearing mice (**Figures 5B-D**). Additionally, a significant amount of serum IL-15 was detected in the mice treated with B7H3-sIL15-NK cells (**Figure 5E**). Seven days after adoptive cell transfer, the frequency and number of CAR-NK cells in the blood of mice treated with either mbIL-15 or sIL-15 were significantly higher (*p* < 0.001) than those in the NK cell and B7H3-NK cell groups, and there was no notable difference (*p* = 0.954) between mbIL-15 and sIL-15 groups. However, even at doses of 5×10^6^ or 2×10^6^ cells, sIL-15 engineering caused uncontrollable proliferation of CAR-NK cells and ultimately lead to premature death of mice. Notably, CAR-NK cells co-expressing mbIL-15 showed moderate cell proliferation and peaked at day 28 and thereafter gradually declined, leaving only a small amount CAR-NK cells in the blood by day 63 after injection (**Figure 5F**, **Supplementary Figure 3**). Therefore, as compared to sIL-15, ectopic expression of mbIL-15 improved CAR-NK cell proliferation moderately and thus therapeutic efficacy without observable side-effects.

### 3.5 mbIL-15 maintained efficacy while eliminating systemic toxicity

**Figure 6:**
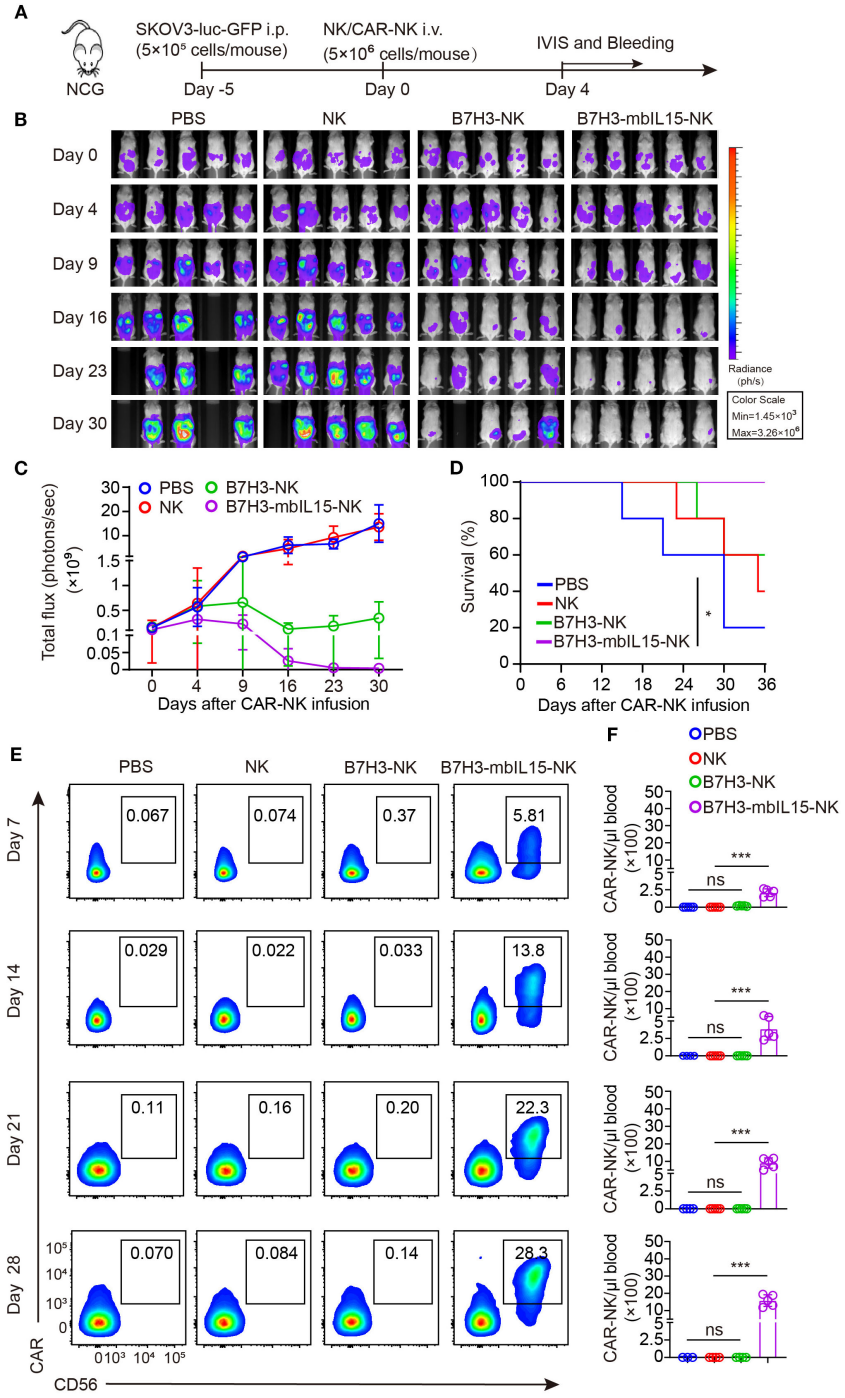
B7H3-NK cells co-expressing mbIL-15 show enhanced tumor control and improved survival when systemically administered. **(A)** Schematic diagram of intravenously administrated 5×10^6^ NK, B7H3-NK, B7H3-sIL15-NK or B7H3-mbIL15-NK cells therapy in a ovarian cancer xenograft model. **(B)** Bioluminescent monitoring of luciferase-labeled SKOV-3 cells. n=5 mice for each group. **(C)** Quantification of the bioluminescence data shown in **(B)**. **(D)** Kaplan-Meier survival curve of mice in **(B)**. **(E, F)** Proportion and number of CAR-NK cells in mouse blood were assessed by flow cytometry on days 7, 14, 21, and 28 following intravenous injection of 5×10^6^ NK, B7H3-NK or B7H3-mbIL15-NK cells. **(E)** Representative flow cytometry results of CAR-NK cell proportion in mouse peripheral blood. **(F)** Statistical graph of CAR-NK cell number in mouse peripheral blood. One-way analysis of variance was used for comparisons among multiple groups. Survival analysis was performed using the log-rank (Mantel-Cox) test. The data expressed as means ± SD. ns: not significant, * *p* < 0.05 and *** *p* < 0.001.

CAR-NK cells co-expressing mbIL-15 were proved that could effectively eliminate tumor cells when intraperitoneally injected. To further investigate the safety of CAR-NK cells co-expressing mbIL-15 when administrated systemically, ovarian tumor-bearing mice were randomly divided into four groups and received intravenously injection of PBS, non-transduced NK cells, B7H3-NK, or B7H3-mbIL15-NK cells at the dosage of 5×10^6^ per mouse respectively. Bioluminescence was used to monitor the tumor burden at scheduled timepoints for 30 days (**Figure 6A**). The results demonstrated that intravenous injection a single dose of non-transduced NK cells had minimal effect on tumor growth and engineering with B7H3 targeting CAR significantly improved tumor control by NK cells. However, B7H3-NK cells only transiently inhibited the progression of ovarian cancer because of their limited persistence. In contrast, B7H3-mbIL15-NK cells successfully eliminated tumors (**Figures 6B, C**) and prolonged the survival of mice (**Figure 6D**). At the same time, peripheral blood of the mice was collected weekly to monitor the frequency and number of CAR-NK cells using flow cytometry. Notably, when intravenously administrated, the co-expression of mbIL-15 maintained the long-term survival and stable expansion of CAR-NK cells without signs of dysregulated growth (**Figures 6E, F**), showing similar performance to intraperitoneal injection strategy.

### 3.6 Safety assessment of CAR NK cells co-expressing mbIL-15 *in vivo*

**Figure 7:**
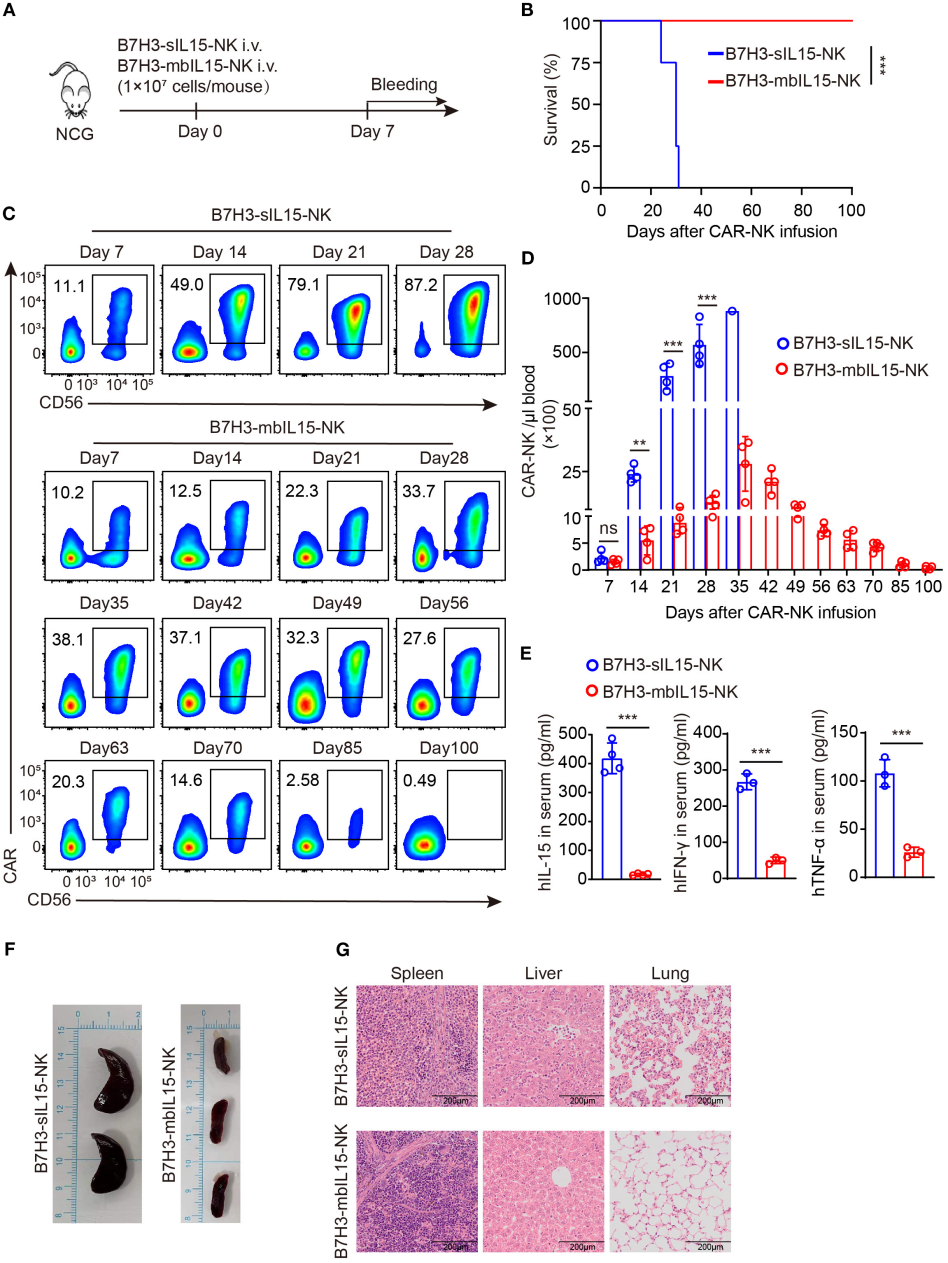
Evaluation of the safety of B7H3-mbIL15-NK cells *in vivo*. **(A)** Schematic diagram of the safety assessment following intravenous administration of 1×10^7^ B7H3-sIL15-NK or B7H3-mbIL15-NK cells in non-tumor-bearing mice. n=5 for each group. **(B)** Kaplan-Meier survival curve of non-tumor-bearing mice. **(C, D)** Proportion and number of CAR-NK cells in non-tumor-bearing mouse blood were assessed by flow cytometry on days 7, 14, 21, 28, 35, 42, 49, 56, 63, 70, 85, and 100 following intravenous injection with 1×10^7^ B7H3-sIL15-NK or B7H3-mbIL15-NK cells. **(C)** Representative flow cytometry results of CAR-NK cell proportion in mouse peripheral blood. **(D)** Statistical analysis of CAR-NK cell number in mouse peripheral blood. **(E)** Human IL-15, IFN-γ and TNF-α concentrations in the plasma of mice at the end of the experiment was measured by ELISA. **(F)** Photographs of the spleens of mice from the groups of B7H3-sIL15-NK or B7H3-mbIL15-NK cells at the end of the experiment or at the point of imminent death. **(G)** Representative Hematoxylin and Eosin (H&E) staining images of the spleen, liver, and lungs from mice in the groups of B7H3-sIL15-NK or B7H3-mbIL15-NK cells at the end of the experiment. Differences between two independent samples were assessed using an unpaired Student’s t-test. One-way analysis of variance was used for comparisons among multiple groups. Survival analysis was performed using the log-rank (Mantel-Cox) test. The data expressed as means ± SD. ns: not significant, *** *p* < 0.001.

To assess the safety of CAR-NK cells co-expressing mbIL-15 *in vivo*, 1×10^7^ of B7H3-sIL15-NK cells or B7H3-mbIL15-NK cells were injected into tumor-free mice via the tail vein and monitored for over 100 days (**Figure 7A**). Even in the absence of tumor antigen stimulation, CAR-NK cells co-expressing sIL-15 caused lethal toxicity, resulting in the death of mice. In contrast, the mice treated with CAR-NK cells arming with mbIL-15 showed no significant side effects, and survived until the end of the experiment (**Figure 7B**). Throughout the experiment, the frequency and number of CAR-NK cells in the blood was detected weekly by flow cytometry. The results showed that CAR-NK cells co-expressing sIL-15 or mbIL-15 had similar frequencies and numbers at day 7 after infusion. However, CAR-NK cells co-expressing sIL-15 continued to proliferate rapidly since the second week after injection (**Figures 7C, D**). In contrast, CAR-NK cells co-expressing mbIL-15 maintained a relatively stable expansion trend, reaching a peak in frequency and number on day 35 after injection, and then gradually declined. By the end of the experiment, only a small number of CAR-NK cells could be detected in the blood of B7H3-mbIL15-NK injected mice (**Figures 7C, D**).

In addition, a significant elevation (*p* < 0.001) of human IL-15, IFN-γ and TNF-α in the serum of mice injected with B7H3-sIL15-NK cells was detected (**Figure 7E**). Finally, we found that mice injected with B7H3-sIL15-NK cells had abnormally enlarged spleens, severe lung inflammation, and liver pathology damage and a significant population of CAR-NK cells was detected in spleen. In contrast, CAR-NK cells co-expressing mbIL-15 did not cause significant major organs damage (**Figures 7F, G**, **Supplementary Figure 4**). In summary, ectopic expression of mbIL-15 in CAR-NK cells was much safer than overexpression of sIL-15.

## 4 Discussion

This study demonstrated that co-expressing of sIL-15 causes autonomous proliferation of CAR-NK cells, resulting in severe toxic side effects and rapid mortality in mice. Conversely, co-expressing mbIL-15 supports long-term survival and stable cell growth *in vivo*. Both intraperitoneal or intravenous administration of CAR-NK cells armoring with mbIL-15 significantly inhibited tumor progression, extended the survival of tumor-bearing mice without causing observable side effects.

Herein, human peripheral blood mononuclear cells (PBMCs) were used as the source for CAR-NK cells. In human peripheral blood, 85%-90% of NK cells are CD56^dim^ subtype, which express numerous activating receptors and effector molecules, allowing them to rapidly recognize and kill target cells with high cytotoxicity (21, 22). However, NK cells are much less frequent in human peripheral blood than T cells (23, 24). Currently, NK cells are usually expanded on a large-scale using feeder cells. However, this method poses a potential safety risks and required irradiation before reinfusion into patients to inhibit the propagation and viability of feeder cells (25). In this study, NK cells were efficiently expanded from PBMCs *ex vivo* using a feeder-cell-free expansion method. This approach requires only a small amount of peripheral blood (40~50 mL) and yield NK cells with high purity (over 90%) (**Supplementary Figures S5A, B**), sufficient for clinical therapeutic dose (2~10 × 10^9^ cells) (**Supplementary Figure S5C**). This significantly reduces the production cost of CAR-NK cells products and enhances their safety in clinical application. Compared to NK cells freshly isolated from peripheral blood, *ex vivo*-expanded NK cells express higher levels of natural cytotoxicity receptors (NCRs) NKp30, NKp44, NKp46, and activating receptor NKG2D (**Supplementary Figures S5D, E**). Furthermore, the CAR transduction efficiency of NK cells is relatively lower than that of T cells, possibly due to their innate resistance to viral vectors (26–28). By optimizing the transduction protocol, CAR constructs were stably and efficiently expressed on the surface of expanded NK cells, transduction efficiency exceeding 80% while preserving the activity and proliferation of NK cells.

NK cells have a shorter lifespan, which results in CAR-NK cells providing temporary therapeutic effects in clinical treatment. Combining systemic supplementation of cytokines, particularly IL-2, has proven to be an effective strategy for enhancing NK cell persistence (29). Systemic administration exogenous human IL-2 can effectively prolong the survival of adoptively transferred NK cells (30, 31). IL-15 and IL-2 have similar structures and both belong to the helical cytokine family. The heterotrimeric receptor of IL-15 shares the IL-2R/IL-15Rβ (CD122) and the common γc chain (CD132) with the IL-2 receptor (32). Because of their shared receptors and similar intracellular signaling JAK1/JAK3/STAT5 pathways, IL-15 and IL-2 share biological activities, including enhancing the proliferation and effector functions of NK cells (33). Clinical studies have confirmed that IL-15 have minimal toxic side effects compared to IL-2, without severe adverse reactions such as capillary leak syndrome (34).

These findings suggest that combining IL-15 could improve the persistence and antitumor activity of CAR-NK cells *in vivo*. Currently, various forms of IL-15 are being researched for NK-based therapy (15). Notably, soluble IL-15 has a short half-life, of less than 1 hour in mice and between 2.5 and 12 hours in patients (35). Frequent and continuous administration of IL-15 makes dosage control difficult and may cause adverse reactions such as CRS, neurotoxicity, and even autoimmune diseases (36, 37). N-803, a novel IL-15 super-agonist, consisting of an IL-15 mutant bound to a short IL-15Rα and human IgG1-Fc. This complex mimics the physiological trans-presentation of IL-15, boasts superior pharmacokinetic properties (38). Nevertheless, reports have demonstrated that frequent subcutaneous injection of N-803 cause inflammatory toxicities such as rash and fever (39, 40).

Incorporating the IL-15 gene into the CAR construct could improve CAR-NK cell survival capacity, anti-apoptotic ability, mitochondrial function, and cytotoxicity (41, 42). A clinical trial using CD19.CAR-NK cells expressing the IL-15 gene to treat B-cell malignancies achieved positive results. Of the 11 patients treated, four with lymphoma and three with CLL achieved complete remission. Furthermore, the ectopic production of IL-15 greatly enhanced the persistence of CAR-NK cells. Remarkably, cord blood-derived CAR-NK cells remained detectable in the blood 12 months after infusion (19). The success of this treatment regimen motivated us to develop CAR structures that co-expressing IL-15 to enhance the persistence and efficacy of CAR-NK cells for solid tumors through ectopically produced IL-15.

In this study, we prepared B7H3.CAR-NK cells co-expressing sIL-15 and validated their function. We found that co-expression of sIL-15 not only enhanced CAR-NK cell proliferation and cytotoxicity *in vitro* and *in vivo*, but also resulted in excessive production of IL-15 in mice, leading to uncontrolled CAR-NK cell proliferation. The dysregulated growth of CAR-NK cells and elevated human IL-15 induced severe inflammatory responses. These included weight loss, diarrhea, hunching, and hypothermia, which led to significant pathological damage in major organs. Reducing the dosage to one-fifth of the original amount only slightly delayed the mice’s death and could not prevent severe toxic side effects. A study revealed that anti-CD123 CAR-NK cells, which secreting IL-15, successfully inhibited leukemia progression but also caused severe systemic toxicity (20). Mice received this therapy rapidly exhibited clinical signs like weight loss, hunching, and death, which were consistent with our findings. Furthermore, similar symptoms occurred when non-tumor-bearing mice were injected with CAR-NK cells co-expressing sIL-15, indicating that the toxicity was independent of mouse models. Anchoring IL-15 onto the surface of NK cells may be alternative strategy to avoid the systemic secretion of IL-15. Tethered IL-15 has been shown to enhance the antitumor activity of tumor-specific T cells and increase the proportion of stem cell memory subsets. No abnormal growth or malignant transformation of T cells was observed in the animal experiments (43).

Herein, we designed a gene sequence that encodes a functional membrane-bound IL-15 and inserted it into a B7H3 targeting CAR construct to ensure sustained expression of IL-15-IL15-Rα on CAR-NK cell surface. *In vitro* experiments demonstrated that co-expression of mbIL-15 enhanced CAR-NK cell proliferation and antitumor activity, comparable to the effects of sIL-15 co-expression. In animal studies, CAR-NK cells armed with mbIL-15 demonstrated great antitumor activity without observable side-effects. These results confirmed that mbIL-15 could potentially address the persistence and safety challenges associated with CAR-NK cell therapy.

CAR-NK cells co-expressing mbIL-15 significantly suppressed tumor growth, however, they did not completely eradicate tumors in the intraperitoneal ovarian cancer xenograft model. At the end of the experiment, CAR-NK cells were still detectable in the blood of mice, but over 60% of them exhibited minimal residual tumor. A recent study suggested that CAR-NK cells injected intravenously may transfer into a dysfunctional state after repeated stimulation with tumor cells, characterized by impaired metabolic adaptation and high proportion of inhibitory receptors. CAR-NK cells that losing their metabolic competency were virtually unable to compete with tumor cells for nutrients, making tumor recurrence more easily. This research substantiated that secondary infusion might increase the number of functional CAR-NK cells and delay the onset of treatment resistance (44).

## 5 Conclusion

In summary, our findings suggest that co-expressing mbIL-15 is a safe and effective strategy to boost the antitumor activity and persistence of CAR-NK cells. Consequently, our experimental data provide a valuable reference for the clinical application of CAR-NK cell immunotherapy.

## Data Availability

The original contributions presented in the study are included in the article/**Supplementary Material**. Further inquiries can be directed to the corresponding author.
