# Supplementary material for: Signaling intact membrane-bound IL-15 enables potent anti-tumor activity and safety of CAR-NK cells
Source: Front Immunol. 2025 Sep 30;16:1658580. doi: 10.3389/fimmu.2025.1658580 (PMC12518233; doi:10.3389/fimmu.2025.1658580)

Supplementary Material


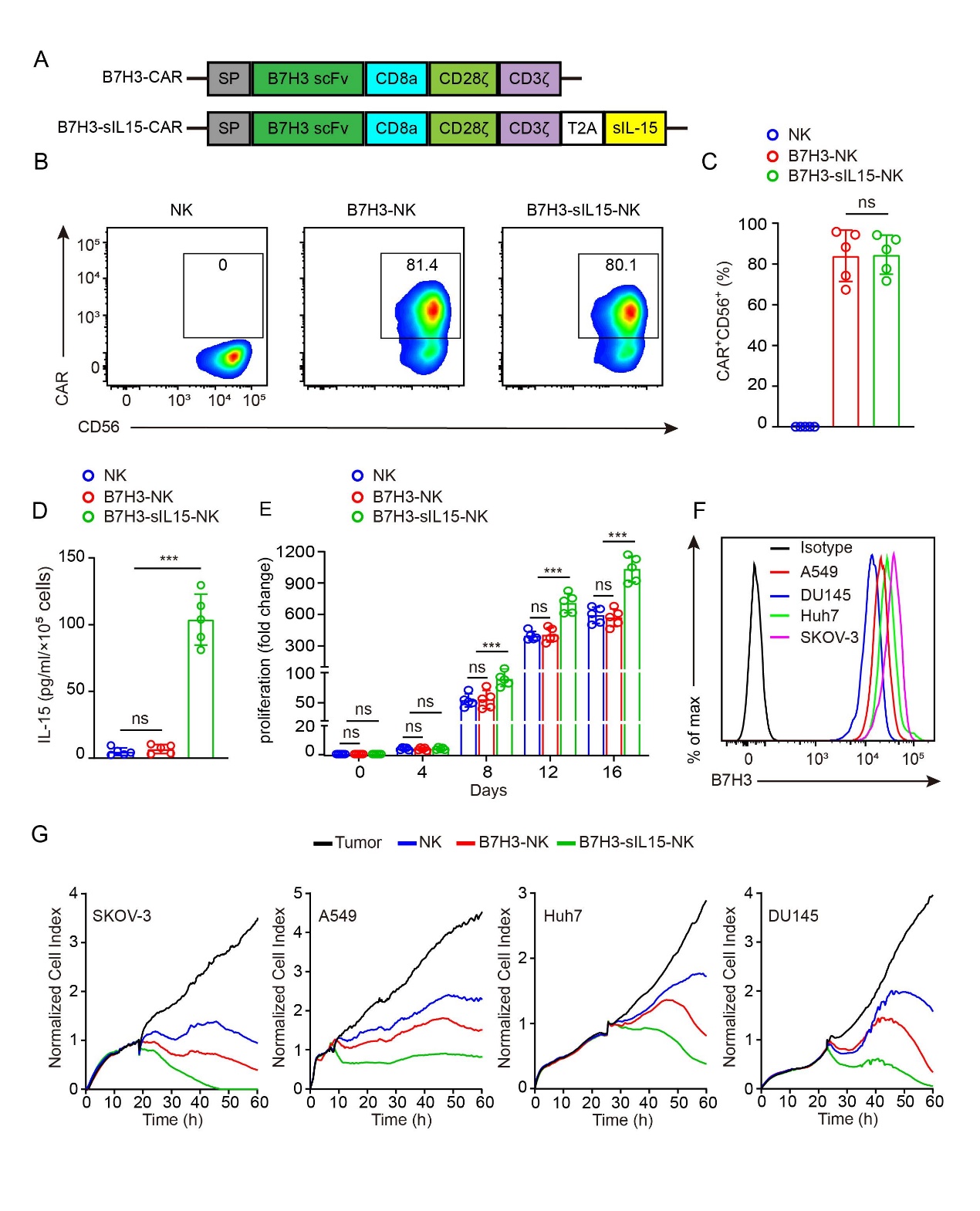


**Supplementary Figure 1. sIL-15 promotes the expansion and enhances the antitumor activity of B7H3-NK cells in vitro**. (A) Structural schema of the B7H3-CAR and B7H3-sIL15-CAR. (B) CAR expression in B7H3-NK or B7H3-sIL-15-NK cells was measured by flow cytometry. (C) Summary of CAR transduction efficiency (n=5). (D) Secretion of IL-15 in the supernatant of 1 × 10⁵ NK, B7H3-NK, or B7H3-sIL15-NK cells was measured by ELISA (n=5). (E) Fold expansion of B7H3-NK or B7H3-sIL15-NK cells was assessed by cell counter (n=5). (F) B7H3 expression in A549, DU145, Huh7 and SKOV-3 cells was analyzed by flow cytometry. (G) Cytotoxicity of NK, B7H3-NK, and B7H3-sIL15-NK cells against B7H3^+^ tumor cells (SKOV-3, Huh7, DU145, and A549 cells) at an E:T ratio of 1:8 was assessed using RTCA. One-way analysis of variance was used for comparisons among multiple groups. The data expressed as means ± SD. ns: not significant, *** P < 0.001.


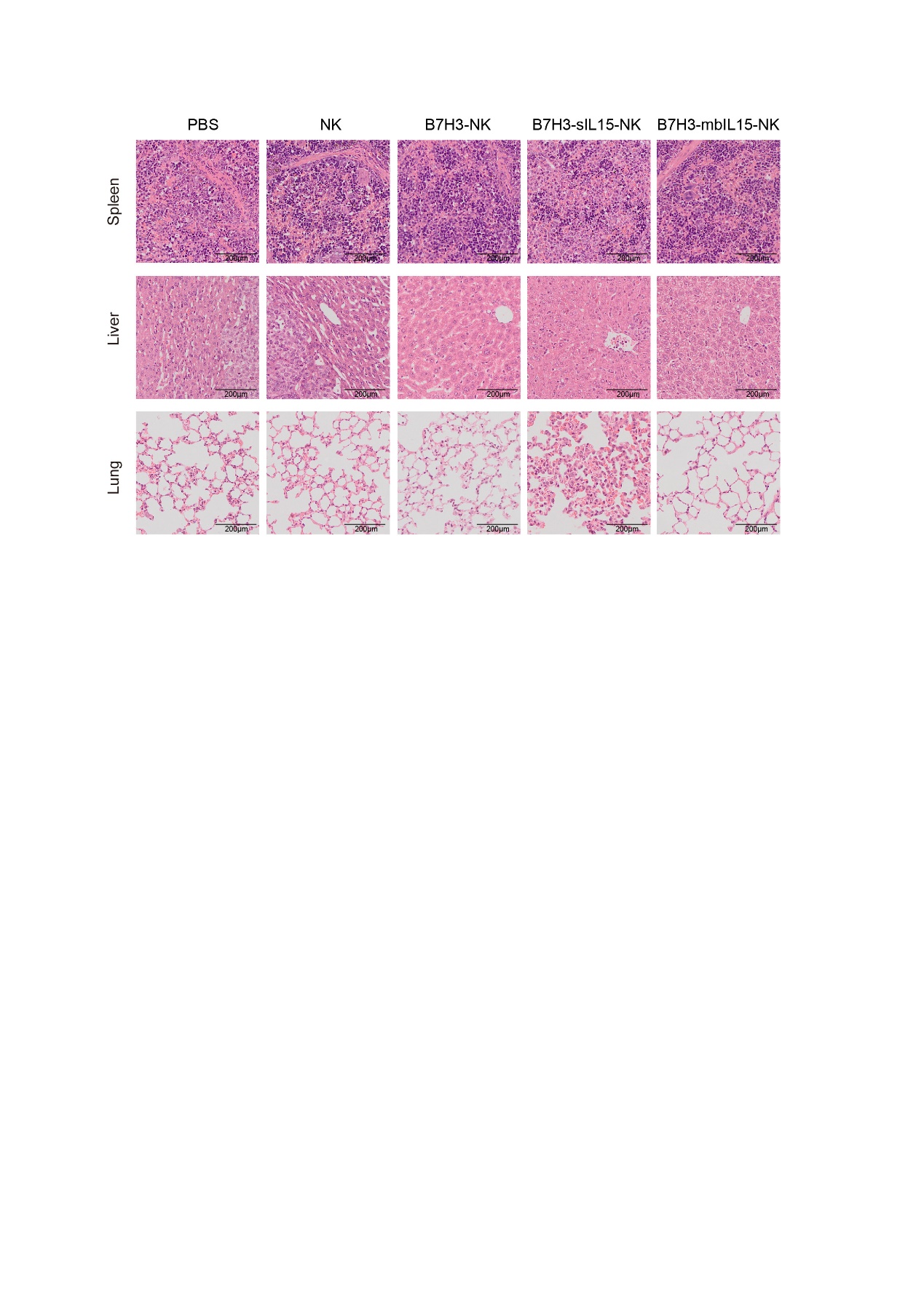


**Supplementary Figure 2.** Representative Hematoxylin and Eosin (H&E) staining images of the spleen, liver, and lungs from mice in the intraperitoneal injection groups of NK, B7H3-NK, B7H3-sIL15-NK or B7H3-mbIL15-NK cells at the end of the experiment.


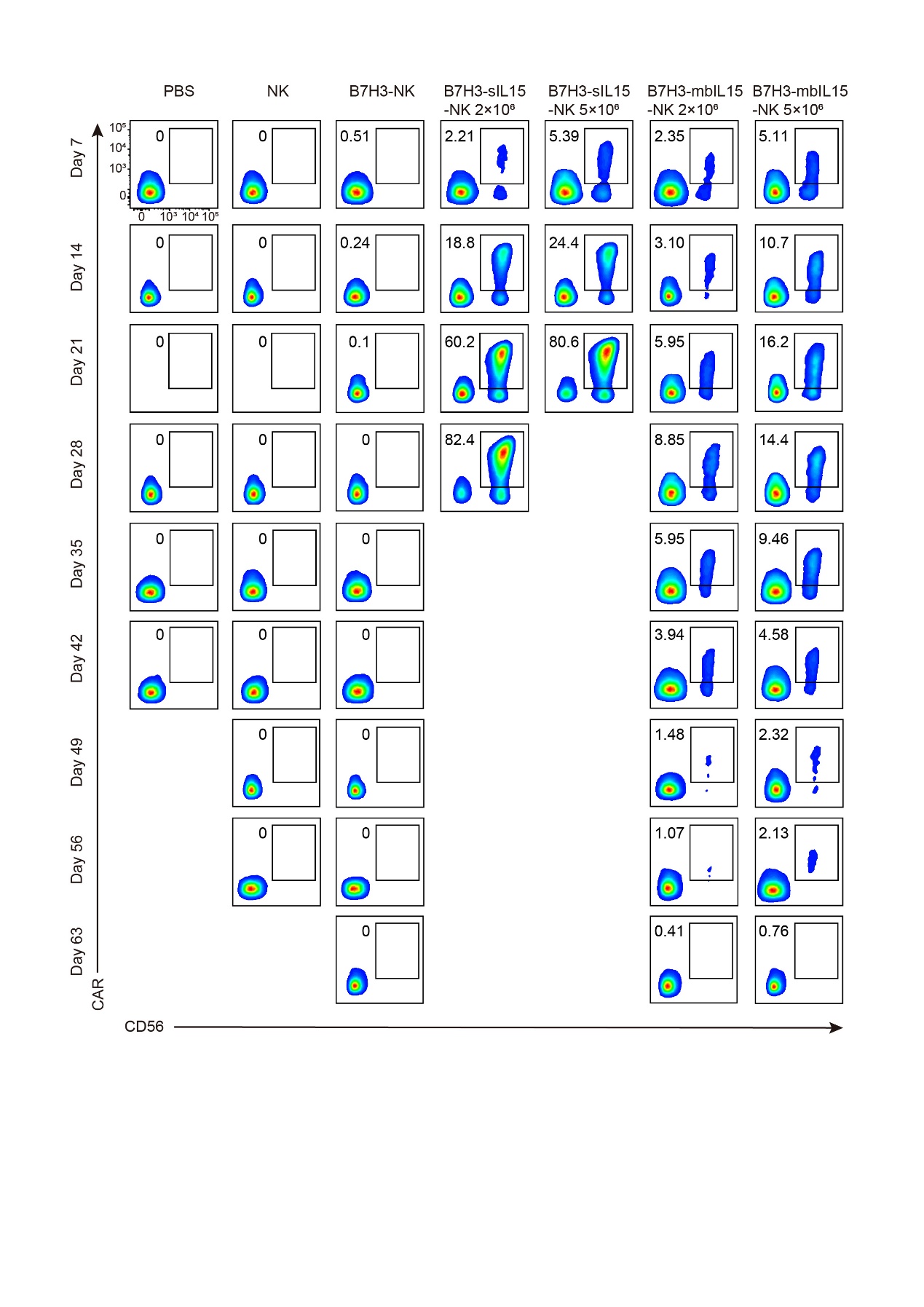


**Supplementary Figure 3.** Representative flow cytometry of CAR-NK cell proportion in mouse peripheral blood. Proportion of CAR-NK cells in mouse blood were assessed by flow cytometry on days 7, 14, 21, 28, 35, 42, 49, 56 and 63 following intraperitoneal injection with 5 × 10^6^ NK, 5 × 10^6^ B7H3-NK 5 × 10^6^ or 2 × 10^6^ B7H3-sIL15-NK and 5 × 10^6^ or 2 × 10^6^ B7H3-mbIL15-NK cells.


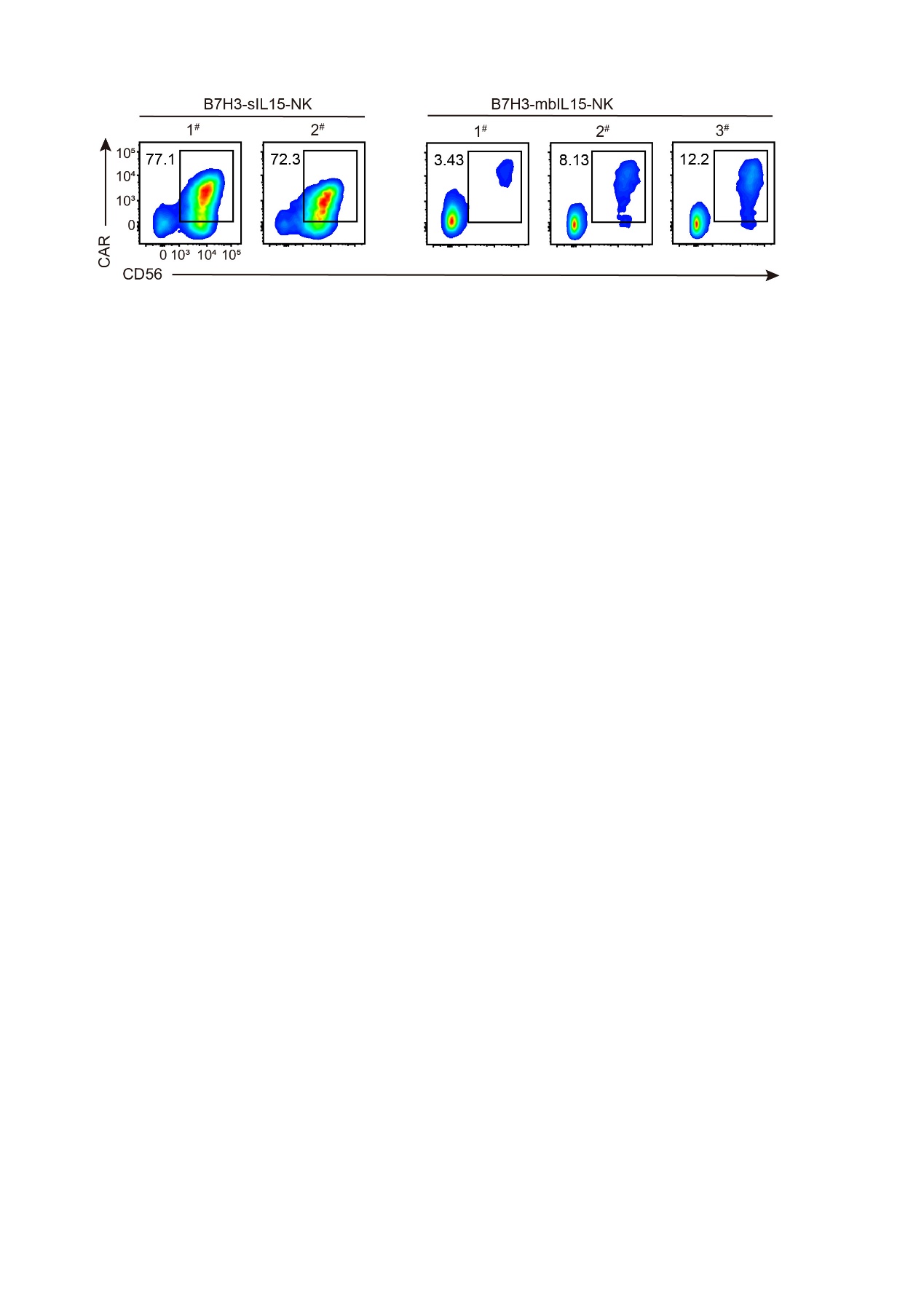
 **Supplementary Figure 4.** Representative flow cytometry of CAR-NK cell proportion in mouse spleen. Proportion of CAR-NK cells in spleen (related to figure 7F) was assessed at the end of the experiment or at the point of imminent death by flow cytometry.


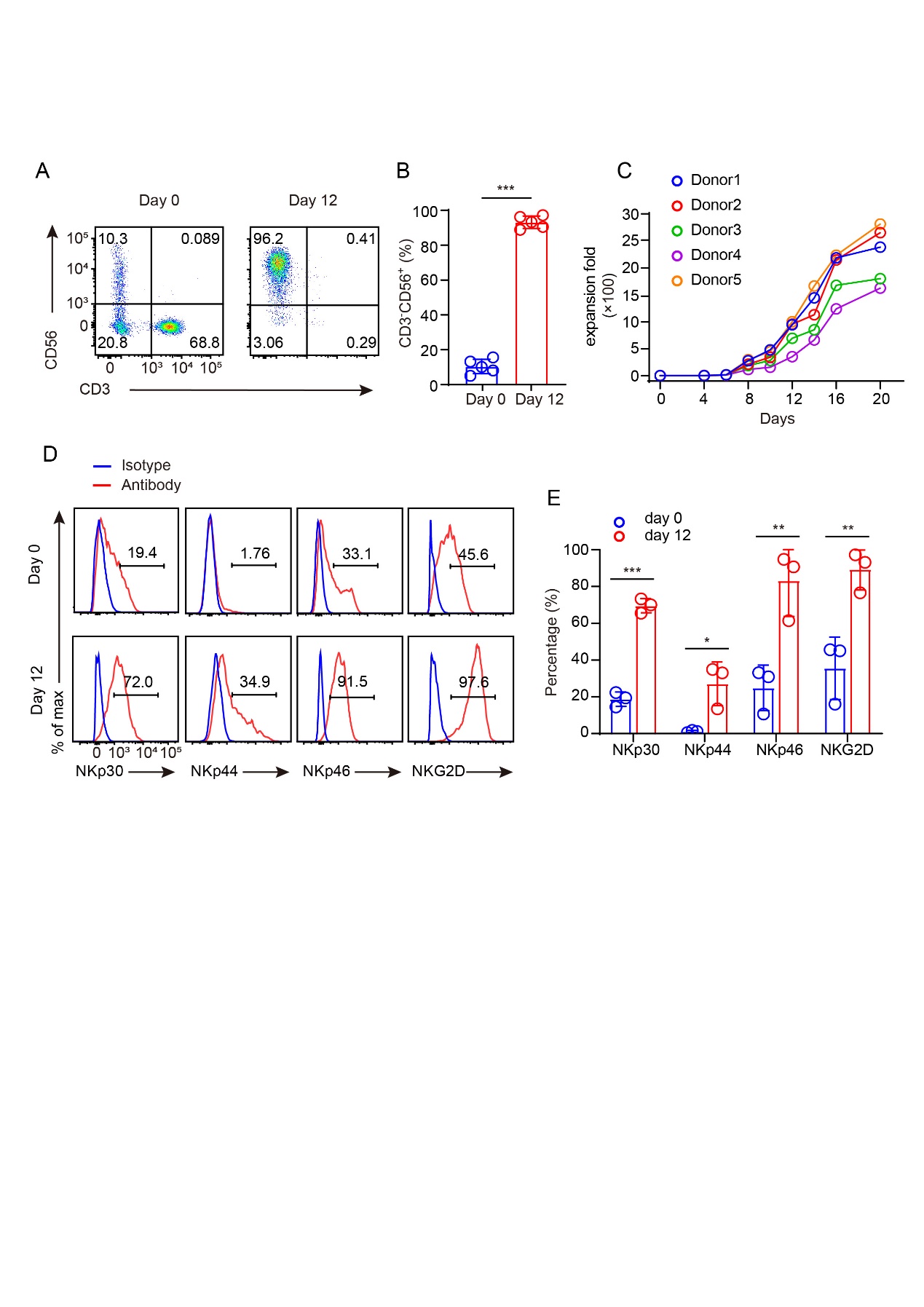


**Supplementary Figure 5. NK cells efficiently expanded in vitro.** (A) shows the representative percentage of NK cells (CD3^-^ CD56^+^) before culture (day 0) and after culture (day 12) using flow cytometry. (B) Statistical analysis of the results shown in (A). (C) The kinetics of NK cells growth during the expansion culture from five healthy donors. (D) The expression of NKG2D and activating NK receptors (NKp30, NKp44, and NKp46) on the corresponding NK cells (gated on CD3^-^ CD56^+^) before culture (day 0) and after culture (day 12) were analyzed by flow cytometry. (E) Statistical analysis of the results shown in (D). Differences between two independent samples were assessed using an unpaired Student’ s t-test. The data expressed as means ± SD. ns: not significant, * P < 0.05, ** P< 0.01 and *** P< 0.001.


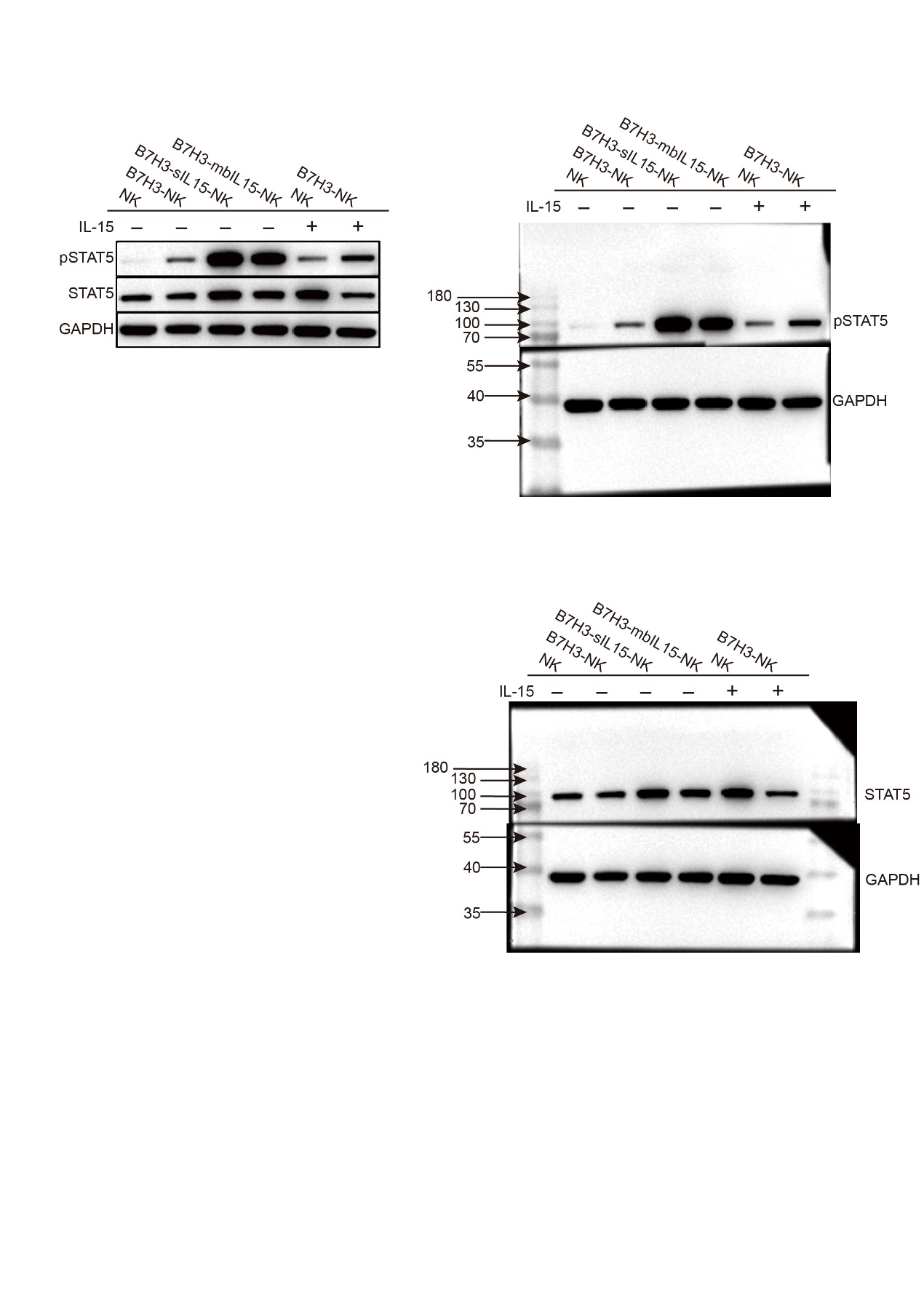

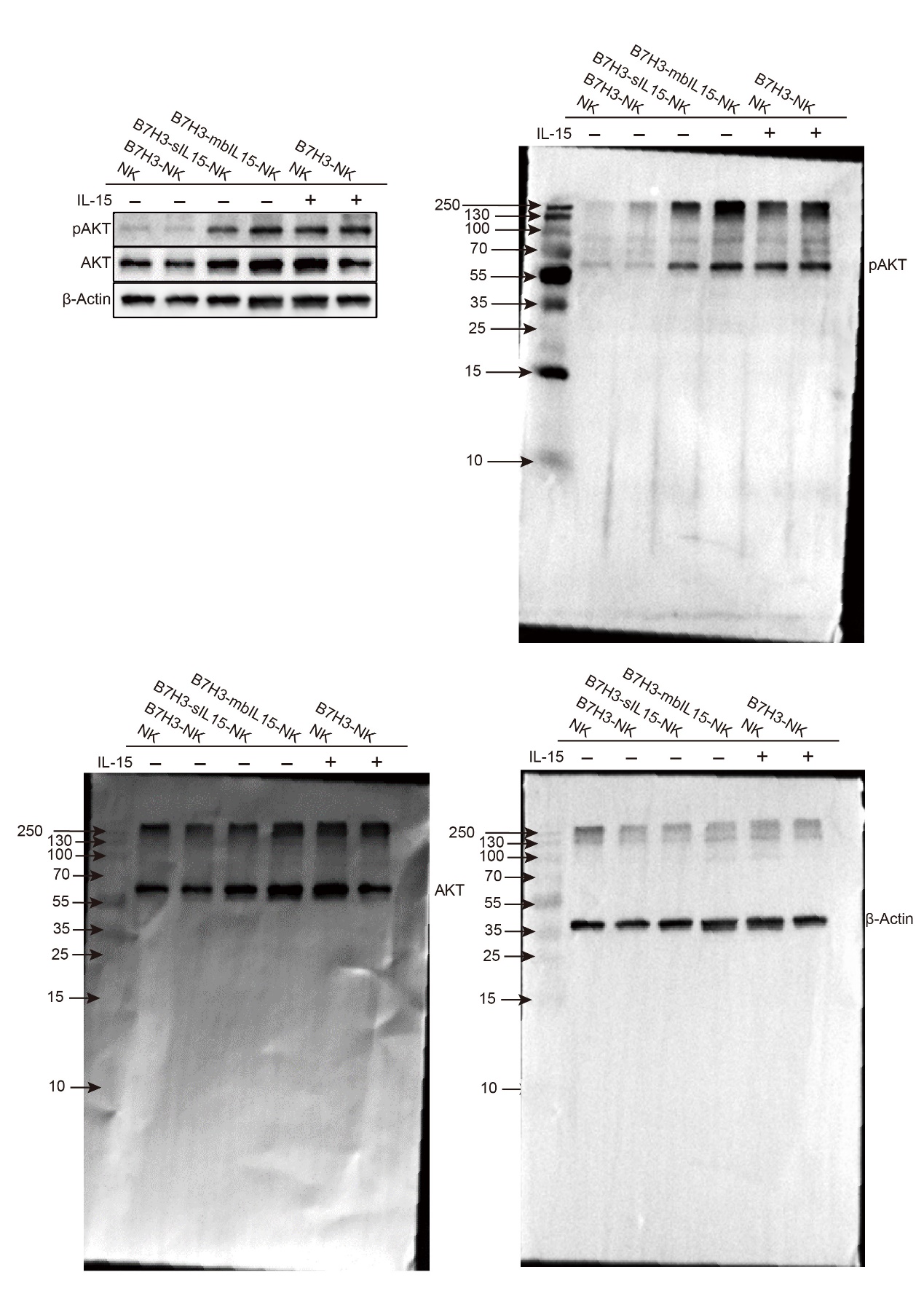

Supplement: Supplementary file 1 [file DataSheet1.docx]
